# Caffeine Consumption among Various University Students in the UAE, Exploring the Frequencies, Different Sources and Reporting Adverse Effects and Withdrawal Symptoms

**DOI:** 10.1155/2022/5762299

**Published:** 2022-05-18

**Authors:** Zelal Kharaba, Nour Sammani, Samar Ashour, Rose Ghemrawi, Ahmad Z. Al Meslamani, Ahmad Al-Azayzih, Manal Ali Buabeid, Yassen Alfoteih

**Affiliations:** ^1^College of Pharmacy, Al Ain University, Abu Dhabi, UAE; ^2^AAU Health and Biomedical Research Center, Al Ain University, Abu Dhabi, UAE; ^3^Department of Clinical Pharmacy, Faculty of Pharmacy, Jordan University of Science and Technology, Irbid, Jordan; ^4^Department of Clinical Sciences, College of Pharmacy and Health Sciences, Ajman University, Ajman, UAE; ^5^Department of Dental Surgery, City University College of Ajman, Ajman, UAE

## Abstract

**Background:**

Caffeine is widely consumed among students due to its cognitive and physical enhancing effects. However, little is known about the consumption pattern of different caffeinated products among university students in the United Arab Emirates (UAE).

**Aim:**

To investigate the frequency of caffeine consumption among the young population of students, assess types of caffeinated products consumed, and document adverse effects and withdrawal symptoms experienced by university students.

**Methods:**

A cross-sectional study was conducted in the UAE from December 2019 to March 2020. A random sample of 500 university students from different universities in the UAE were approached and asked to complete a self-administered online-based questionnaire. Data were analyzed using the Statistical Package for Social Science (SPSS) version 26.

**Results:**

Of (*n* = 500) surveyed students, (*n* = 467) completed the survey 93.4%. The average level of caffeine consumption was significantly higher in females compared to male students (*p* < 0.005). Coffee was the highest favored source of caffeine (67.7%) followed by tea (47.3%). The average daily intake of caffeine was found to be 264 mg/day. Surprisingly, almost a third of students reported a high level of daily consumption (>400 mg/day) and more than half of them consumed less than 199 mg/day. Large proportions of students 91.1% have their caffeinated beverage after or while eating meals and 42.8% considered that this habit helped in avoiding acid reflux. Interestingly, around one third of participants have poor knowledge of caffeine-containing medical products, which seemed to affect the level of consumption in the student population (*p* < 0.05). The highest reported reason for caffeine intake was for studying purposes (59.4%).

**Conclusion:**

Caffeine consumption is highly prevalent among university students in the UAE. Yet, there is insufficiency in the current knowledge of safe caffeine consumption patterns reflecting the importance of health awareness programs and nutritional lectures to decrease the long-term health issues and unintentional overdose of caffeine.

## 1. Introduction

Caffeine, a methylxanthine derivative, is a widely used psychoactive and cognitive enhancer substance globally [1]. In contrast to other psychoactive drugs, it is legal and available in most regions of the world. Within an hour of oral ingestion, caffeine is absorbed and distributed to all body parts, even to the placenta and blood-brain barrier [2]. It exerts various pharmacological and physiological effects on muscles, cardiovascular, renal, and respiratory systems [3]. These effects include positive inotropic and chronotropic effects on the heart, mild diuresis and many others [4]. The most prominent mechanism that explains the stimulatory activity of caffeine appears to be the antagonism of adenosine receptors in the brain, which normally cause drowsiness when activated. Consequently, caffeine promotes wakefulness, the ability to maintain intellectual activity and an improved hedonic tone [2, 5]. Apart from the psychostimulant effects of caffeine, available evidence suggests that coffee consumers are less likely to develop diabetes, coronary heart disease and stroke compared to non-coffee consumers [6]. However, conflicting results from other studies were reported [7]. For instance, a study by Liu et al. found a positive relationship between caffeine intake and all-cause mortality in the population of 55 years old and above [8].

According to the literature, there has been a global increase in caffeine intake over the past decade. In 2020/2021, individuals consumed approximately 166 million, 60 kilograms bags of coffee globally. Several reasons for caffeine consumption have been previously discussed in the literature. Its usage by surgeons to reduce fatigue [9], by athletes to enhance their performance [10], and by students to improve concentration and wakefulness [11].

Students from school and college are heavily taking caffeine nowadays. Seventy-three percent of children consume caffeine daily as stated by the journal Pediatrics by the AAP in 2014 [12]. Most of them drink soft drinks in high amounts and despite the sugar and unhealthy ingredients of these drinks, caffeine in itself can harm their health. Many studies reported various harmful effects of caffeine such as panic attacks, insomnia and chronic fatigue on students. In 2014, an 18-year-old teenager in Ohio, USA, had cardiac arrhythmia and died from an overdose of pure caffeine powder [13].

While many studies revealed similar trends in caffeine intake among students in the Gulf and the Middle East, only limited studies in the UAE highlighted this concern [14, 15]. Therefore, our study aims to investigate the frequency of caffeine consumption among the young population of students, assess types of caffeinated products consumed, and document adverse effects and symptoms of caffeine consumption experienced by university students in the UAE.

## 2. Methods

### 2.1. Study Design, Sampling Technique, And Size

This was a quantitative, cross-sectional study with a descriptive design conducted over four months (from December 2019 to March 2020) on a random sample of university students across the UAE. The study population included females (*n* = 304) and males (*n* = 163) who were 18 years old and above and consented/agreed to participate in the study. The study received the required ethical approvals from the research ethics committee (REC) at Al-Ain University (AAU-REC-B3, Feb 2019). Convenience sampling was used as a technique to research participants.

Based on previous literature and the Raosoft sample size calculator [16], a minimum sample size of 385 students was considered representative for this study.

### 2.2. Study Tool

The questionnaire used in the study is a pre-validated survey. All study participants were administered this survey individually through an online link with the help of the research team who tried to reach students from different majors and universities across the seven emirates. The participants were briefly informed about the study objectives at the beginning of the questionnaire. The survey was administered in English and Arabic. It included closed-ended questions that contained four sections: Characteristics of the sample and demographics, Caffeine consumption patterns, Caffeine expectations, and social expectations and purposes of caffeine consumption.

### 2.3. Characteristics of the Sample and Demographics

The following information was addressed: Age, gender, college, and year of study.

### 2.4. Caffeine Consumption Patterns

Participants answered Yes/No questions about their caffeine consumption behaviors, their knowledge about the existence of caffeine in some medicinal products and their feedback about the use of caffeine in reliving their headaches. This section also addressed the following: When they usually take their caffeinated drink, do they prefer to drink it on an empty stomach or after their meals and if so, why do they choose after meals? What sources of caffeine do they usually consume the most, and from where do they prefer to get it?

### 2.5. Caffeine Expectations

Participants answered four Likert-type statements on their expectations of consuming caffeine. The Likert scale was a 5-point scale of “Highly disagree” stated as number 1, to “Highly agree” stated as number 5 on the scale. In addition, some questions were about caffeine addiction, withdrawal symptoms, and side effects. Caffeine addiction was considered as taking more than 100 mg of caffeine daily, which is about one cup of coffee. The students were given the 1 to 5 scale to report their caffeine consumption to determine their addiction level.

### 2.6. Social Expectations and Purposes of Caffeine Consumption

Participants were asked to answer questions about the purpose of consuming caffeinated products. The questions included a range of reasons and social habits of drinking coffee and other caffeinated products.

### 2.7. Validation of the Study Questionnaire

The questionnaire used for the study is pre-validated. Nevertheless, we tailored it to suit the aims of the study and the culture of the UAE. Another validation test was conducted for the edited version of the questionnaire. A draft of the questionnaire was prepared and sent to a panel of experts who were professors in the Pharmacy and Nutrition field at Al-Ain University, Ajman University and UAE University to test the content validity of the questionnaire. They examined many factors of the questionnaire including the conciseness, length, clarity, language, time consumed, bias, and appropriateness of questions. Content validation of a questionnaire should consider these factors [17].

### 2.8. Reliability Testing of the Study Questionnaire

The questionnaire was additionally revised based on a reliability test conducted as a pilot study on 84 students to achieve the most acceptable Cronbach's values. Also, a preliminary pilot testing was carried out to ensure the practicality and understandability of the questionnaire.

### 2.9. Inclusion and Exclusion Criteria

University students of both genders aged >18 years and currently studying in any university in the UAE were included in the study. The exclusion criteria were students who do not consume caffeine.

### 2.10. Data Collection

The final version of the questionnaire was administered through an online link sent via different social media methods to students who met the eligibility criteria and agreed to participate. The research team carried out the questionnaire distribution. Participants were first briefed about the estimated time needed to complete the survey and the study purposes; they were then directed to the questionnaire link. Furthermore, the participants were informed about the confidentiality and anonymity policy.

### 2.11. Statistical Analysis

Data sets were gathered by the principal investigator and the excel sheet of the online survey was downloaded and then imported to the Statistical Package for Social Science (SPSS) version 26 (IBM Corporation, Armonk, NY, USA) for data analysis. Descriptive statistical analysis was conducted and results were represented as numbers (*n*) with percentages (%). To assess differences in responses across categorical variables, Chi-square or Fisher exact tests were used as appropriate. A *p* value of <0.05 was considered to be statistically significant.

## 3. Results

Four hundred sixty-seven participants completed the questionnaire, of which 65.1% (*n* = 304) were females and more than half were between 20 and 23 years old (Table 1).

The majority of the participants (38.5%) reported that they consume caffeine two to three times daily, and around 7% reported a consumption rate of more than 5 times per day. The vast majority of participants (90.1%) consume caffeinated drinks with or after meals. Roughly a third of them (34.7%) attributed this behavior to their knowledge of caffeine consequences on the stomach i.e. acid reflux and stomach upset, while more than half (51%) consider it a habit. Around 316 (67.7%) picked coffee as their favorite drink, 221 (47.3%) picked tea, and 172 (36.8%) picked chocolate (Table 2).

Additionally, there were significant differences in favorite drinks across females and males. Females were more likely to prefer coffee and chocolate, whereas males were more likely to prefer tea (*p* < 0.005). On the other hand, there was no significant difference in caffeine consumption between the medical and non-medical colleges (*p*=0.149).

More than half of students (56.3%) agreed that the combination of caffeine and analgesic medications is more effective in relieving headaches. However, more than two-thirds of students (66.4%) had significantly poor knowledge about caffeine's existence in medical products and this seemed to influence their caffeine consumption rate (*p* < 0.05) (Table 3).

The findings of this study indicated that withdrawal symptoms and side effects, such as muscle pain, shakiness, and trouble concentrating were significantly associated with the age of students (*p* values < 0.05). Interestingly, more than 30% of participants reported caffeine use more than 3 times a day at the age of 8–12 years, and more than 40% reported the same frequency at the age of 13–17 years (Table 4). When further exploring the expectation of caffeine consumption, we found that more than half of the participants thought it allowed them to think clearly, sharpen their memory and improve their mood (Table 5).

**Figure 1:**
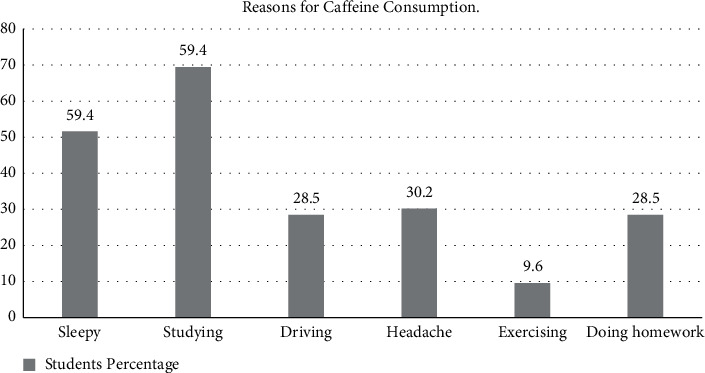
Reasons for caffeine consumption (expressed as %).

More than half of the students (59.4%) reported consuming caffeine for studying and avoiding sleep (Figure 1). Lastly, we compared the average caffeine consumption per day and gender among participants and found that 28.7% (*n* = 134) consumed >400 mg/day which is considered a high consumption level. However, there was no difference in daily consumption among medical and non-medical students or gender (Table 6).

## 4. Discussion

Little is known about caffeine's safe and healthy consumption behaviors, even though there is a rise in coffee intake by the young population. A limited number of studies conducted in the Gulf region and the UAE reported the prevalence of caffeine consumption among university students. Yet and to the best of our knowledge, no other study assessed types of caffeinated products consumed with the documentation of adverse effects and withdrawal symptoms experienced by university students. Therefore, our current study aimed to evaluate the consumption pattern of different caffeinated products among university students in the UAE, reporting caffeine's adverse effects and withdrawal symptoms and characterizing its consumption with age, gender and other characteristics. Finally, we sought to determine the motivational factors behind student's consumption of various caffeinated beverages.

Our study results indicated that more than half of the students consume less than 199 mg/day, while almost a third of the students reported a high level of daily consumption (>400 mg/day). The average daily intake of caffeine was found to be 264 mg/day. These findings were in agreement with a previous study of caffeine consumption in the UAE [18] and Bahrain universities (268 mg/day) [19]. However, the average daily consumption in our study sample was lower compared to students from Lebanon (373 mg/day during the weekdays) [20] and higher compared to caffeine intake among U.S college students who reported consuming average daily caffeine of 173 mg/day [11]. Similar results of average daily caffeine consumption in gulf universities might represent the similar habits and behaviors in these countries.

Female students were found to consume more caffeinated beverages than males, with a daily average of 275 mg and 242 mg, respectively. This might be due to different lifestyles and hangouts of female students compared to males in the Arabic world. However, this finding opposes the results of a study conducted among college students of Zayed University, UAE [15], where no significant difference was reported between genders. The discrepancy between the results could be attributed to the large number of participants included in our study.

Coffee was the most commonly consumed source of caffeine by respondents, followed by tea and caffeinated soft drinks. Coffee being the primary source of caffeine, was similar to previous reports. However, according to these studies, soft drinks preceded tea consumption [19]. These results can reflect the social view of coffee as more prestigious than other drinks and thus increasing their usage trend among students in the UAE.

Forty percent of the students take caffeinated products two to three times daily and a quarter of them take it only one time, preferably in the early morning. Fortunately, more than two-thirds of the sample population take caffeinated beverages after a meal; upon asking them about the reason, half of them follow this practice to avoid stomach upset and enhance digestion. However, a study of coffee consumption among Saudi female students by H. Alfawaz mentioned that 18.7% of the students suffering from stomach pain was considered to be related to coffee consumption [21].

Our results indicated that most of the surveyed students consume caffeinated beverages to enhance their alertness and concentration while studying; sharpen their memory, and improve their mood as represented by Figure 1. A study from Korea referred reported that the motivation for caffeinated drink consumption was due to various reasons such as social factors, alertness, health, mood, daily habits as well as sensory effects. The motivation of consumption was different and depended on the source of caffeine and different kinds of caffeinated drinks [22].

The surveyed participants have reported various symptoms associated with their high caffeine consumption, such as higher levels of tension and anxiety, difficulty falling asleep, and rapid heart rate. While it is not easy to link these symptoms to the high level of caffeine consumption, many studies have shown a strong association between such symptoms and caffeine consumption [22–24]. Reports from Australia and Korea showed that caffeine consumption and energy drink intake was correlated with the poor quality of sleep among adults [25, 26]. In contrast, other reports showed no correlation between caffeine consumption and poor quality of sleep [27].

Interestingly, more than 30% of participants reported the caffeine use more than 3 times a day at the age of 8–12 years and more than 40% at the age of 13–17 years. In fact, a study published in 2010 answered the question of why should we be worried about caffeine consumption in children [28]. The results of several studies indicated that caffeine could have different effects on children and adolescents compared to those seen in adults. Indeed, childhood and adolescence is a period of speedy growth; and proper nutrition and sleep are considered essential to maximize this growth and development. Many studies concluded that caffeine use disrupts sleep patterns [28, 29]. In addition, results from animal studies indicated that caffeine could prime the brain to increase its response to subsequent drug exposure, thereby potentiating the reinforcing effects of drugs [30]. Children and adolescents can be susceptible to these effects, especially since their brains are still undergoing significant development. Thus, the results of our studies are of significance to the academic community as it increases the awareness of caffeine consumption among this population and encourage the implementation of educational platforms and workshops to educate the young population and parents about caffeine consumption among students.

## 5. Conclusion

Caffeine consumption is prevalent among university students in UAE (93.6%). Our study highlights the lack of knowledge of safe caffeine consumption patterns among these students and hence reflect the importance of health awareness programs and nutritional lectures to decrease the long-term health issues and unintentional overdose of caffeine. Our results can be considered baseline data to be utilized in further research about coffee consumption trends in the Arab world.

## Figures and Tables

**Table 1:** Demographics of students (*n* = 467).

	*N* (%)
*Total*	467 (100)

*Gender*	
Male	163 (34.9)
Female	304 (65.1)

*Age*	
17–19	93 (19.9)
20–23	263 (56.3)
24–27	65 (13.9)
28+	46 (9.9)

*College*	
Medical	201 (43)
Non-medical	266 (57)

*Year of Study*	
First year	63 (13.5)
Second year	93 (19.9)
Third year	112 (24)
Fourth year	124 (26.6)
Fifth year	75 (16.1)

Descriptive analysis was conducted.

**Table 2:** Caffeine consumption patterns.

*How often do you consume caffeinated beverages? N (%)*	
Once daily	116 (24.8)
2-3 times a day	180 (38.5)
4-5 times daily	72 (15.4)
More than 5 times a day	32 (6.9)
1 to 3 times weekly	50 (10.7)
4 to 6 times weekly	17 (3.6)

*What time of the day do you usually consume caffeine* ^*∗*^	
Morning	284 (60.8)
Afternoon	187 (40.0)
Evening	218 (46.7)

*When do you prefer to have caffeine?*	
Before eating	171 (36.6)
While eating	106 (22.7)
After eating	302 (67.4)

*Why do you prefer having your caffeine drink with/after meals?*	
To help with digestion	90 (19.3)
Habit	238 (51.0)
To avoid acid reflux and stomach upset	162 (34.7)

*Sources of caffeine* ^*∗*^	
Coffee	316 (67.7)
Tea	221 (47.3)
Caffeinated soft drinks	124 (26.6)
Energy drinks	77 (16.5)
Chocolate	172 (36.8)

*Do you know that caffeine is added to certain pain killers\medicines?*	
No	157 (33.6)
Yes	310 (66.4)

*Does a cup of coffee with Panadol relieve your pain/headache more effectively than Panadol alone?*	
No	204 (43.7)
Yes	263 6.3)

^*∗*^Multiple responses allowed. Descriptive analysis was conducted.

**Table 3:** Average consumption level.

Average caffeine consumption level		1 to 3 times weekly	2-3 times a day	4 to 6 times weekly	4-5 times daily	More than 5 times a day	Once daily	*p*-value
Parameters	Total							
*Gender*								
Female	304	40	130	14	26	16	78	**0.005**
Male	163	10	50	3	46	16	38	

*College*								
Medical	201	22	12	50	80	27	10	0.149
Non-medical	266	28	5	66	100	45	22	

*When did you start taking caffeine (years)*								
13 to 17	210	27	92	9	31	9	42	0.153
8 to 12	77	4	28	2	16	6	21	
Over 18	148	18	48	6	22	13	41	
Under 8	32	1	12	1	2	4	12	

*Are you aware that caffeine is added to certain pain relievers?*								
Yes	310	27	116	13	54	29	71	**0.005**
No	157	23	64	4	18	3	45	

Chi-square or fisher exact tests were used as appropriate.

**Table 4:** Caffeine side effects and withdrawal symptoms.

Parameter	*N* (%)	At what age did you start consuming caffeine? (Years)				*p*-value
		8 to 12	13 to 17	Over 18	Under 8	
*Withdrawal symptoms*						
Headaches	175 (37.47)	22 (12.57)	81 (46.28)	61 (34.85)	11 (6.28)	0.359
Nausea	42 (8.99)	13 (30.95)	13 (30.95)	12 (28.57)	4 (9.52)	0.074
Muscle pain	37 (7.92)	14 (37.83)	9 (24.32)	10 (27.02)	4 (10.81)	**0.003**
Drowsiness	127 (27.19)	15 (11.81)	62 (48.81)	42 (33.07)	7 (5.51)	0.185
Fatigue	61 (13.06)	4 (6.55)	43 (47.77)	24 (39.34)	6 (9.83)	
Irritability/Poor mood	90 (21.41)	17 (18.88)	19 (28.78)	23 (25.55)	7 (7.77)	0.677

*Side effects*						
Shaky	66 (14.13)	15 (22.72)	26 (41.93)	24 (36.36)	8 (12.12)	**0.040**
Tense	62 (13.27)	15 (24.19)	61 (48.41)	17 (27.41)	4 (6.45)	0.512
Rapid heartbeat	126 (26.98)	19 (15.07)	20 (4.28)	38 (30.15)	8 (6.34)	0.864
Anxious	61 (13.07)	14 (22.95)	9 (20.45)	22 (36.06)	5 (3.27)	0.310
Trouble concentrating	44 (9.42)	11 (25.00)	23 (43.39)	17 (38.63)	7 (15.90)	**0.004**
Restless	53 (11.34)	8 (15.09)	48 (41.02)	18 (33.96)	4 (7.54)	0.985
Difficulty falling asleep	117 (25.05)	14 (11.96)		44 (37.60)	11 (9.40)	0.202

Bold indicates a significant result. Findings are listed as numbers. Chi-square or fisher exact.

**Table 5:** Caffeine expectations.

		Strongly disagree (%)	Disagree (%)	Neutral (%)	Agree (%)	Strongly agree (%)
Caffeine makes me	Alert, energized and less sleepy	6.9	9.6	25.7	28.9	28.9
	Think clearly, pay more attention and sharpen my memory	3.9	8.8	25.7	38.8	22.9
	Relax, calm down, and improves my mood	4.9	7.5	24.6	35.3	27.6

Descriptive analysis was conducted.

**Table 6:** Caffeine average consumption.

Caffeine consumers	% (*n*)
Low (≤199 mg/day)	52.0% (*n* = 243)
Moderate (200–399 mg/day)	19.3% (*n* = 90)
High (>400 mg/day)	28.7% (*n* = 134)

*Caffeine average consumption per day and gender*	
Male	242.3 mg
Female	275.1 mg

*Caffeine average consumption per day and college*	
Medical	279.7 mg
Non-medical	251.5 mg

Descriptive analysis was conducted.

## Data Availability

The datasets used and analyzed during the present study are available from the corresponding author on reasonable request.

## Additional Points

Strengths and weaknesses. The main study limitation is that it's a survey-based study, which can affect the accuracy of reported details. Another limitation is the unfeasibility to calculate the exact amount of caffeine content ingested by students.
